# Supplementary material for: Foxp3 orchestrates reorganization of chromatin architecture to establish regulatory T cell identity
Source: Nat Commun. 2023 Nov 6;14:6943. doi: 10.1038/s41467-023-42647-y (PMC10628232; doi:10.1038/s41467-023-42647-y)
Supplement: Supplementary file 1 — Supplementary Information [file 41467_2023_42647_MOESM1_ESM.pdf]

Inventory of Supplementary Information

Supplementary Figure 1. Flow cytometry isolation of T cell populations in the thymus and spleen

Supplementary Figure 2. CRISPR deletion of Foxp3 sites near the Ikzf2 gene

Supplementary Figure 3. CRISPR/Cas9 deletion of Foxp3 in mature Treg cells

Supplementary Data 1. Hi-C sequencing statistics

Supplementary Data 2. Differential chromatin interactions between Treg and Tcon

Supplementary Data 3. Differential gene expression between WT and KIKO Tregs

Supplementary Data 4. Differential gene expression between WT and DSM Tregs

Supplementary Data 5. Differential Foxp3 Cut&Tag between WT and DSM Tregs

Supplementary Data 6. Foxp3 PLAC-seq significant interactions

Supplementary Table 1. ssDNA sequence for generating Foxp3 DSM mutant mouse

Supplementary Table 2. Antibodies used in this study

Supplementary Table 3. Foxp3/CTCF Binding sites in Ikzf2 locus and related targeted sgRNA sequences

Supplementary Figure Legends

Supplementary Figure 1. Flow cytometry isolation of T cell populations in the thymus and spleen. (a) Flow cytometric gating strategy to isolate T cells in the thymus, including $CD4^{-}CD8^{-}$ (DN), $CD4^{+}CD8^{+}$ (DP), $CD4^{-}CD8^{+}$ (CD8SP), $CD4^{+}CD8^{-}Foxp3^{-}CD25^{-}$ (CD4SP), $CD4^{+}CD8^{-}Foxp3^{-}CD25^{+}$ ($CD25^{+}$ Treg precursor), $CD4^{+}CD8^{-}Foxp3^{lo}CD25^{-}$ ($Foxp3^{lo}$ Treg precursor), $CD4^{+}CD8^{-}Foxp3^{+}CD25^{+}$ (Treg). (b) Flow cytometric gating strategy to isolate $CD4^{+}Foxp3^{-}$ (conventional T cells, Tcon) and $CD4^{+}Foxp3^{+}$ (mature Treg) from the spleen. (c) Heat map showing changes in Treg and Tcon specific chromatin interactions during T-cell lineage development by K-means clustering (k=6). The top panel shows Treg specific interactions, the bottom panel shows Tcon specific interactions. Each row is normalized relative to the mean obs/exp interaction frequency across each lineage.

Supplementary Figure 2. CRISPR deletion of Foxp3 sites near the Ikzf2 gene. (a) Hi-C chromatin interactions near the Ikzf2 gene. The heatmap shows Hi-C data near Ikzf2, while the ChIP-seq tracks below show signals for CTCF and Foxp3 binding. Also labeled are the locations of the 9 deleted Foxp3 sites (P1-P9). (b) Flow cytometric analysis of the expression of Helios after CRISPR knockout of indicated Foxp3-bound sites (Ikzf2 P1-P9) in Treg cells. *p*-values were calculated by an unpaired, two-sided Student's *t*-test. Data are represented as mean ± s.e.m. Representative data of two independent experiments are shown. Source data are provided as a Source Data file.

Supplementary Figure 3. CRISPR/Cas9 deletion of Foxp3 in mature Treg cells. (a) Schematic of experimental strategy for deletion of Foxp3 in mature Treg cells. (b) FACS plots showing sgFoxp3 targeting efficiency in knockdown of Foxp3 expression in Treg cells. *p*-values were calculated by an unpaired, two-sided Student's *t*-test. Data are represented as mean ± s.e.m. Representative data of two independent experiments are shown. Source data are provided as a Source Data file. (c) Comparison of chromatin interactions between sgControl and sgFoxp3 targeted Treg cells. The plot shows the strength of chromatin contacts (X-axis) and the fold change in chromatin interaction frequency (y-axis) between sgControl and sgFoxp3 targeted cells. Only one interacting locus shows a significant difference in interaction frequency (colored in red). (d) Violin plots of fold change in chromatin interaction frequency between mutant and wild type comparing the effects in the Foxp3 KIKO Treg cells (left, orange) and in Foxp3 sgRNA targeted cells (right, blue).

a

Gating strategy for T cells in the thymus

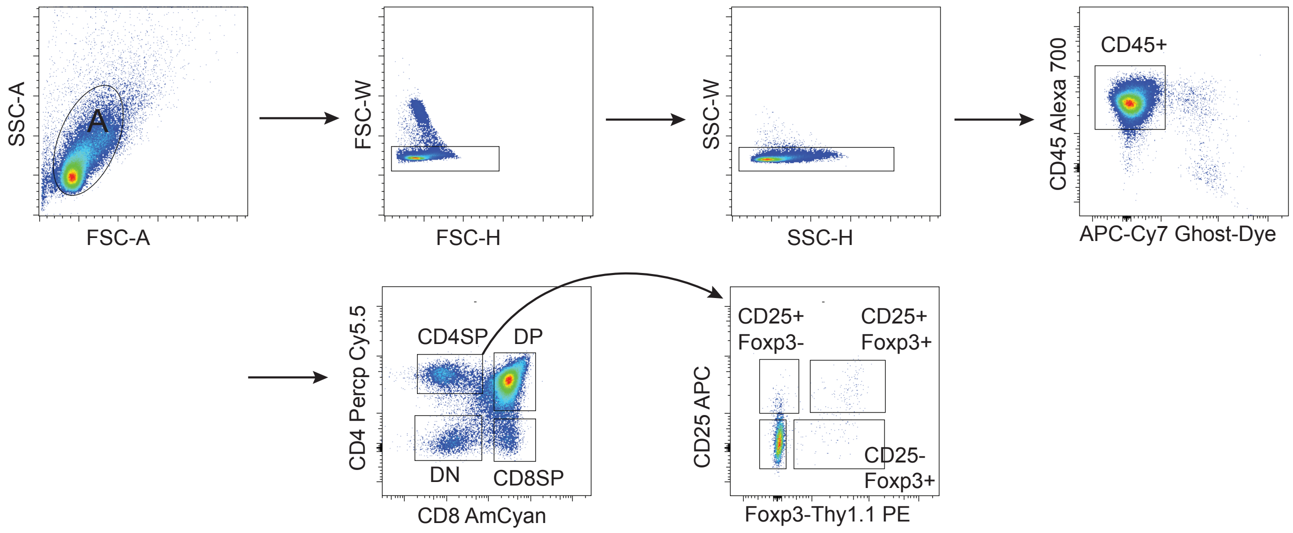

b

Gating strategy for Treg and Tcon cells in the spleen

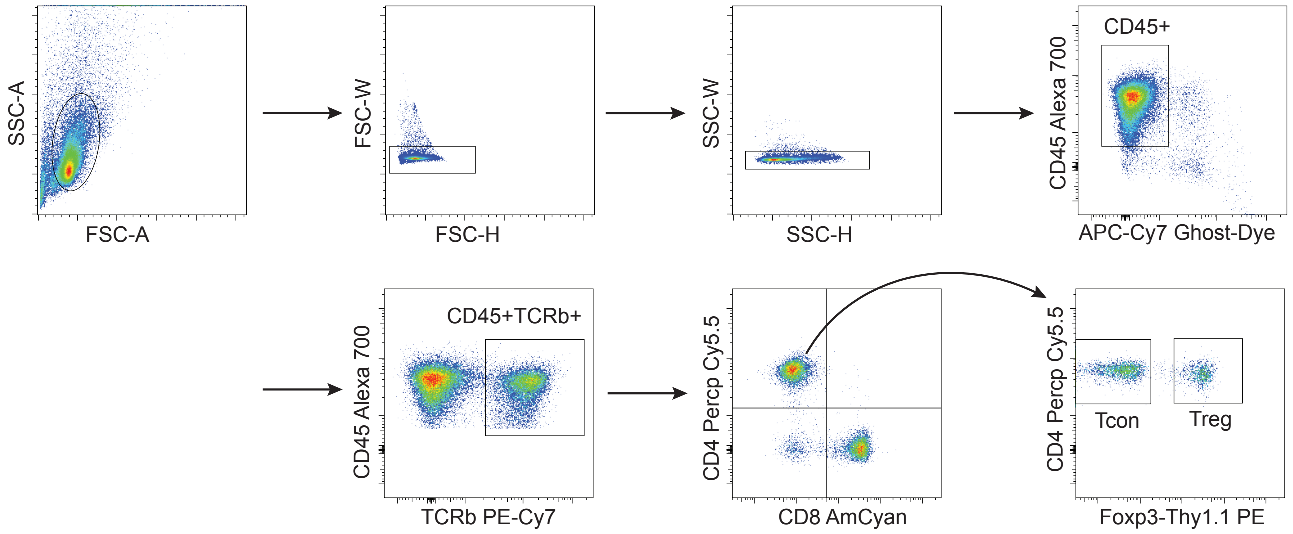

c

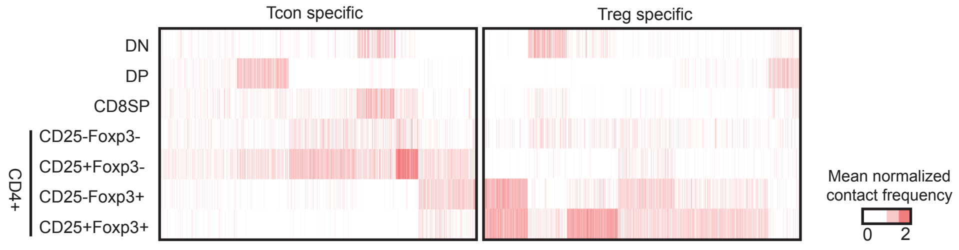

Supplementary Figure 1

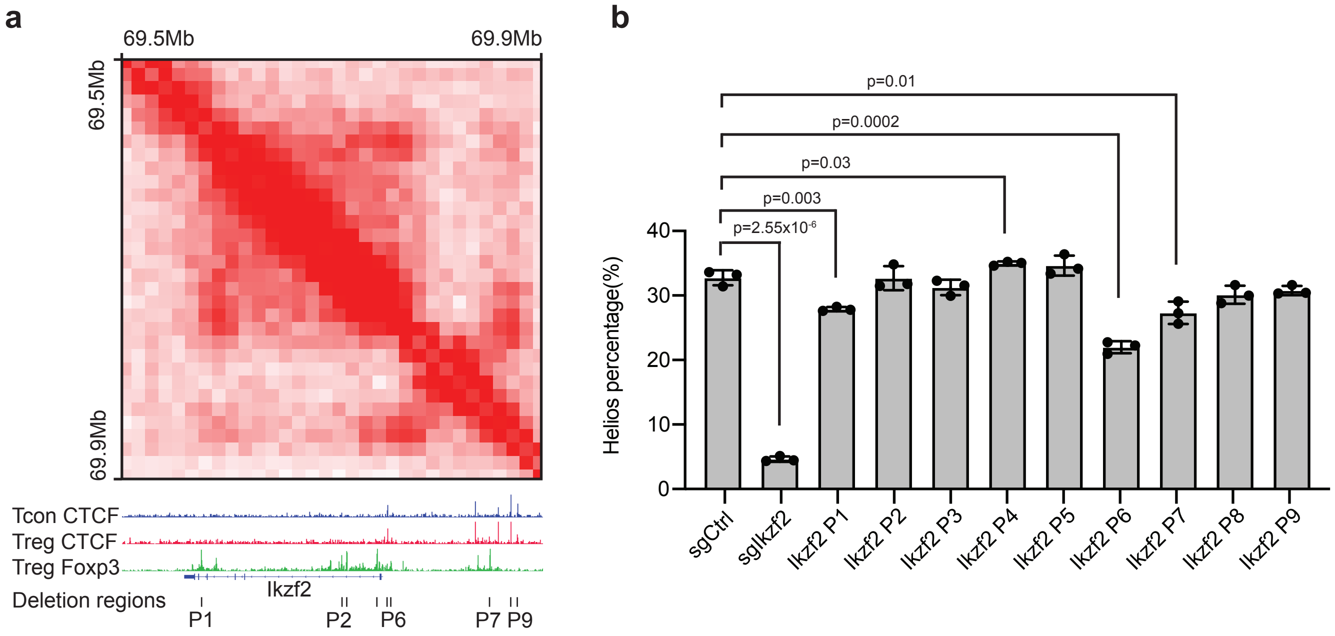

Supplementary Figure 2

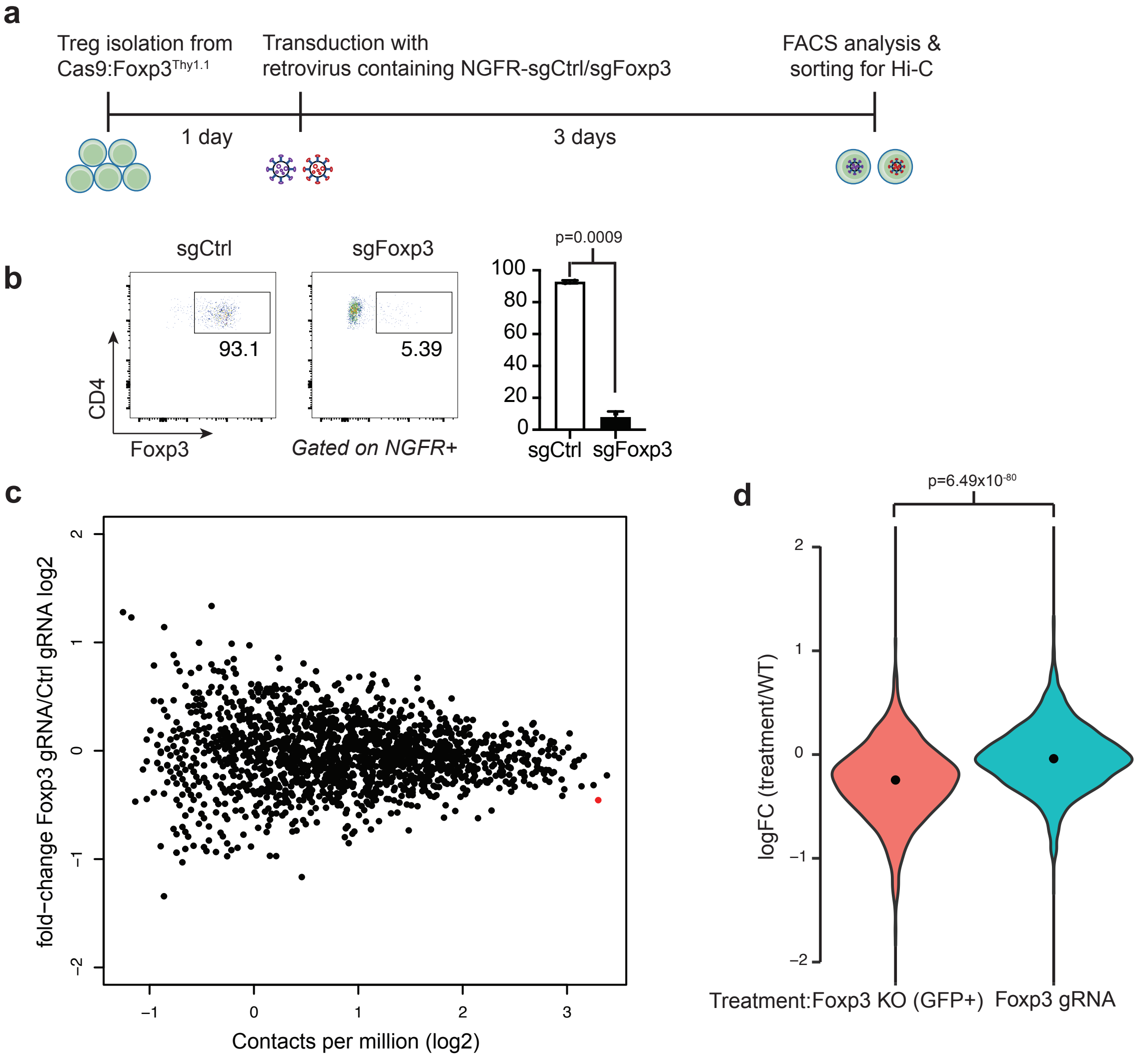

Supplementary Figure 3

Supplementary Table 1, ssDNA sequence for generating Foxp3 DSM mutant mouse

aagtcaggtg cactcaacca agctaaccaa ccctctccca cctgtcaggc ctgggttgtg
agtttaccag ggaccataga tatttggtgt caggctggct atgccacttg agctgcttac
atgcctttga tgtacaaatt acttgactcc tttttaaagt gaggagagct atttggcagg
agtactgcaa agaagacaca gcttacggcg ggtactcagt aaacagtact atgtgtgagc
atagactgtc cctccccccct tggtgctagt ggtaggaatt gagaccttgg attcctgatg
cagacaaagg tggggtaggg ggtgaggagg ccaaaggctc tgatctatgc caaccttctg
cagagttctt ccacaacatg gactacttca agtatcacaa tatgcgaccc cctttcacct
atgccaccct tatccgacag gtaagcaggg caatagaggc ccagcagctg gtgggcggca
gggggggagt tgtggtgggg agtgcttgcc tcctacattg caccaagagc agaattcacc
cattaacaaa cctcagctct gaggagcccc aagatgtgat ccttcttgat agcttcacct
cagatctagc cctcaaccca aaactactgc aagccaggtc agtgcaaagc aaactgtaac
actacaaact accctttcct ttgtccaccc tatctctaac atcacccttg acctcatgcc
tcaccctatt ctttctcctt ccccttgacc cacaattaca aagctatcat agctcagagg
gccgagagta ggctgctccc tcagccacaa ccctgaggaa catgcccctt attccacctg
actccaactt ccaggccatc ctggaagccc cggagaggca gaggacactc aatgaaatct
accattggtt tactcgcact ttcccgtact tcagaaacca ccccgccacc tggaaagtga
gttcctctgt acacactggc agctgggatg gctccaagga tggttagcct ggggctagac
atgtggggaa ggagcaggtc agtctcagac tcaggatgac tgtcaaccct gtccctgact
ggggtcccgg tccccccttcc acagaatgcc atccgccaca acctgagcct gcacaagtgc
tttgtgcgag tggagagcga gaagggagca gtgtggaccg tagatgaatt tgagtttcgc
aagaagagga gccaacgccc caacaagtgc tccaatccct gcccttgacc tcaaaaccaa
gaaaaggtgg gcgggggagg gggccaaaac catgagactg aggctgtggg ggcaaggagg
caagtcctac gtgtacctat

The three DNA codons labeled in red color encode the 3 amino acid mutations in the Foxp3DSM mutant.

Supplementary Table 2, Antibodies used in this study

Target protein	Vendor	Catalog #	clone	dilution
CD45.2-Alexa 700	Biolegend	109822	104	Flow (1:400)
CD45RB-APC	eBioscience	17-0455-81	C363.16A	Flow (1:400)
TCRb-PE-Cy7	eBioscience	25-5961-82	H57-597	Flow (1:400)
TCRgd-PE	BioLegend	118108	GL3	Flow (1:400)
CD3-BV650	BioLegend	100229	17A2	Flow (1:400)
CD4-PerCP-Cy5.5	TONBO	65-0042-U100	RM4-5	Flow (1:400)
CD8-BV510	Biolegend	100752	53-6.7	Flow (1:400)
CD25-FITC	TONBO	35-0251-U100	PC61.5	Flow (1:400)
CD44-BV650	Biolegend	103049	IM7	Flow (1:400)
CD62L-BV605	Biolegend	104438	MEL-14	Flow (1:400)
IFNg-APC	eBioscience	17-7311-82	XMG1.2	Flow (1:400)
IL-5-PE	eBioscience	12-7052-82	TRFK5	Flow (1:400)
Il-13- eflour660	eBioscience	50-7133-82	eBio13A	Flow (1:400)
IL-17-PE	eBioscience	12-7177-81	eBio17B7	Flow (1:400)
human CD2-PE	eBioscience	12-0021-83	RPA-2.10	Flow (1:200)
NGFR-APC	Biolegend	345108	ME20.4	Flow (1:400)
Ghost Viability Dye	TONBO	13-0865-T100		Flow (1:1000)
CTCF	Millipore	07-729		ChIP
Foxp3 Rabbit polyclonal antibody	In house			ChIP

Supplementary Table 3, Foxp3/CTCF Binding sites in Ikzf2 locus and related targeted sgRNA sequences.

ChIP-seq peak	Chromosome coordinates	Assigned region name	gRNA sequence	gRNA targeting location
Treg_Foxp3ChIP_MACS_peak	chr1:69590531-69591726	P1	ACCCCAGAACTCATCTAAGG	chr1:69590510
			AAGGTTTGATGTCTGCCCGG	chr1:69591970
Treg_Foxp3ChIP_MACS_peak	chr1:69699765-69700462	P2	AATAATAATTCTGGTCATGG	chr1:69699360
			ACACATCTTTAACAGTACAG	chr1:69700490
Treg_Foxp3ChIP_MACS_peak	chr1:69703613-69704447	P3	TAAAAACAAATGCTGCGCAG	chr1:69703550
			AATGGGAGCGAAACTGATTG	chr1:69704690
Treg_Foxp3ChIP_MACS_peak	chr1:69726524-69728147	P4	GCTATAAGTATAGAGTGTCT	chr1:69726650
			GGGTGAGAAAGTCCCAGTGT	chr1:69727980
Treg_CTCF_MACS_peak	chr1:69735671-69735970	P5	GCGTATCTGCAAAGTCATGG	chr1:69735550
			TAAACCGTAGCAATTCACGT	chr1:69736110
Treg_Foxp3ChIP_MACS_peak	chr1:69738549-69739093	P6	TGAGATGCCCGATATAGTAT	chr1:69738330
Treg_CTCF_MACS_peak	chr1:69738974-69739155		GGTAATGTGCTAGACACCTG	chr1:69739250
Treg_Foxp3ChIP_MACS_peak	chr1:69815193-69816119	P7	GCTTGAGATGCTGATCCTAG	chr1:69814970
			AACTTGCCGATTGCTACAGG	chr1:69815980
Treg_CTCF_MACS_peak	chr1:69831756-69832019	P8	GGCTTGTTAGCTCTCAAGCA	chr1:69831290
			AATGAATTAGGCATTAGCAG	chr1:69832430
Treg_CTCF_MACS_peak	chr1:69836869-69837145	P9	AAAAGATGCCCTTATCTATG	chr1:69836880
			GCCTTATGTGGGTAGCATGG	chr1:69837400
		sgIkzf2-exon4	ACCCCTAATTGAGAGCAGCG	
