# Supplementary material for: Foxp3 orchestrates reorganization of chromatin architecture to establish regulatory T cell identity
Source: Nat Commun. 2023 Nov 6;14:6943. doi: 10.1038/s41467-023-42647-y (PMC10628232; doi:10.1038/s41467-023-42647-y)
Supplement: Supplementary file 3 — Description of Additional Supplementary Files [file 41467_2023_42647_MOESM3_ESM.pdf]

**Description of Additional Supplementary Files**

**File Name: Supplementary Data 1**
**Description: Hi-C sequencing statistics.** Overall statistics of the Hi-C sequencing data for each T cell sample in the study, including the numbers of raw reads, aligned reads, unique reads, cis chromatin interactions (<1kb and >1kb), trans chromatin interactions, and the percentages of cis and trans chromatin interactions.

**File Name: Supplementary Data 2**
**Description: Differential chromatin interactions between Treg and Tcon.** Table of differential chromatin interactions between Treg and Tcon cells using 25kb bins from edgeR. The table lists the chromosome, starting and ending coordinates of the intering bin anchors, the RPM normalized interaction frequency in Tcon and Treg cells, the log2 fold-change in interaction frequency, the log2 counts-per-million interaction frequency across all samples, the likelihood ratio from the edgeR quasi-likelihood F-test, the p-value from the edgeR quasi-likelihood F-test, and the Benjamini-Hochberg corrected p-value (fdr).

**File Name: Supplementary Data 3**
**Description: Differential gene expression between WT and KIKO Tregs.** Table of differential gene expression between WT and KIKO Treg cells from edgeR. The table lists the Ensembl gene ID, chromosome, gene start, gene end, gene strand, and common gene name for each gene. In addition, the table contains the log2 fold-change in expression, log2 counts per million across all samples, the likelihood ratio from the edgeR quasi-likelihood F-test, the p-value from the edgeR quasi-likelihood F-test, the Benjamini-Hochberg corrected p-value (fdr), and the CPM normalized expression in WT and KIKO Tregs.

**File Name: Supplementary Data 4**
**Description: Differential gene expression between WT and DSM Tregs.** Table of differential gene expression between WT and DSM Treg cells from edgeR. The table lists the Ensembl gene ID, chromosome, gene start, gene end, gene strand, and common gene name for each gene. In addition, the table contains the log2 fold-change in expression, log2 counts per million across all samples, the likelihood ratio from the edgeR quasi-likelihood F-test, the p-value from the edgeR quasi-likelihood F-test, the Benjamini-Hochberg corrected p-value (fdr), and the CPM normalized expression in WT and DSM Tregs.

**File Name: Supplementary Data 5**
**Description: Differential Foxp3 Cut&Tag between WT and DSM Tregs.** Table of differential Foxp3 Cut&Tag data between WT and KIKO Treg cells from edgeR. The table lists the peak coordinates, the log2 fold-change in Cut&Tag signal, log2 counts per million across all samples, the likelihood ratio from the edgeR quasi-likelihood F-test, the p-value from the edgeR quasi-likelihood F-test, and the Benjamini-Hochberg corrected p-value (fdr).

**File Name: Supplementary Data 6**
**Description: Foxp3 PLAC-seq significant interactions.** The table lists the coordinates of enriched Foxp3 binding peaks from PLAC-seq experiments in bedpe format.
