# Supplementary material for: Foxp3 orchestrates reorganization of chromatin architecture to establish regulatory T cell identity
Source: Nat Commun. 2023 Nov 6;14:6943. doi: 10.1038/s41467-023-42647-y (PMC10628232; doi:10.1038/s41467-023-42647-y)
Supplement: Supplementary file 10 — Reporting Summary [file 41467_2023_42647_MOESM10_ESM.pdf]

nature portfolio

Corresponding author(s): Ye Zheng, Jesse R Dixon

Last updated by author(s): Sep 20, 2023

Reporting Summary

Nature Portfolio wishes to improve the reproducibility of the work that we publish. This form provides structure for consistency and transparency in reporting. For further information on Nature Portfolio policies, see our Editorial Policies and the Editorial Policy Checklist.

Statistics

For all statistical analyses, confirm that the following items are present in the figure legend, table legend, main text, or Methods section.

n/a	Confirmed	
☐	☒	The exact sample size (*n*) for each experimental group/condition, given as a discrete number and unit of measurement
☐	☒	A statement on whether measurements were taken from distinct samples or whether the same sample was measured repeatedly
☐	☒	The statistical test(s) used AND whether they are one- or two-sided<br>*Only common tests should be described solely by name; describe more complex techniques in the Methods section.*
☐	☒	A description of all covariates tested
☒	☐	A description of any assumptions or corrections, such as tests of normality and adjustment for multiple comparisons
☐	☒	A full description of the statistical parameters including central tendency (e.g. means) or other basic estimates (e.g. regression coefficient) AND variation (e.g. standard deviation) or associated estimates of uncertainty (e.g. confidence intervals)
☐	☒	For null hypothesis testing, the test statistic (e.g. *F*, *t*, *r*) with confidence intervals, effect sizes, degrees of freedom and *P* value noted<br>*Give P values as exact values whenever suitable.*
☒	☐	For Bayesian analysis, information on the choice of priors and Markov chain Monte Carlo settings
☒	☐	For hierarchical and complex designs, identification of the appropriate level for tests and full reporting of outcomes
☒	☐	Estimates of effect sizes (e.g. Cohen's *d*, Pearson's *r*), indicating how they were calculated

Our web collection on statistics for biologists contains articles on many of the points above.

Software and code

Policy information about availability of computer code

Data collection	FACSDiva v9.0 (BD Biosciences) for flow cytometry.
Data analysis	Hi-C and PLAC-seq data were aligned to the mm9 reference genome using BWA-MEM (v0.7.17). Reads were filtered (MAPQ >= 30) and paired using a previously described pipeline. PCR duplicate reads were removed using Picard (v2.1.1). Contact matrices were generated and normalized using the iterative correction method using Juicer (v1.9.9). Enriched contacts in the PLAC-seq data were identified using HICCUPs in Juicer (v1.9.9). To detect differential chromatin interactions between experiments, we calculated contact frequencies in 25kb bins for all interactions genome wide separated by less than 1Mb. Differential interacting regions were called using edgeR (v3.32.1) with Benjamini-Hochberg correction for multiple testing. Transcription factor motif enrichment in Foxp3-dependent chromatin interaction sites was performed by first identifying differential chromatin interactions between wild-type (Foxp3GFP+) and Foxp3 knockout (KIKO GFP+) Tregs at a resolution of 25kb. Differential interacting regions were overlapped with DNase I Hypersensitive sites in mouse Tregs from ENCODE (accession ENCFF566TDU). Motif enrichment over these DHS sites found in differential interaction regions was performed using Homer (v4.9.1) using the collection of known vertebrate motifs from the JASPAR database<br><br>RNA-seq data and ChIP-seq were aligned by using STAR (v2.6.1) to the mm9 reference genome. PCR duplicates were removed, and read counts were quantified over GENCODE genes (vM1) using HTSeq (v0.13.5) and subject to RPKM normalization. Differentially expressed genes were identified using edgeR (v3.32.1). Gene Ontology analysis was performed using TopGO (v2.42).<br><br>CUT&RUN data was aligned to the mm9 reference genome using BWA-MEM (v0.7.17). Peaks were called using MACS2 (v2.2.7.1). Peaks calls from the Foxp3 experiments together to create a union set of peaks. Using this merged peak set, we then identified differential Foxp3 sites using edgeR (v3.32.1).

Flow cytometry data were analyzed using FlowJo software (10.7.1, BD bioscience).

Statistical analyses were performed using GraphPad Prism v9.0.0 (bar and line graphs).

For manuscripts utilizing custom algorithms or software that are central to the research but not yet described in published literature, software must be made available to editors and reviewers. We strongly encourage code deposition in a community repository (e.g. GitHub). See the Nature Portfolio guidelines for submitting code & software for further information.

Data

Policy information about availability of data

All manuscripts must include a data availability statement. This statement should provide the following information, where applicable:

- Accession codes, unique identifiers, or web links for publicly available datasets
- A description of any restrictions on data availability
- For clinical datasets or third party data, please ensure that the statement adheres to our policy

The sequencing data from the RNA-seq, ChIP-seq, CUT&RUN, Hi-C, and PLAC-seq experiments have been deposited in Gene Expression Omnibus (GEO) with the following accession ID: GSE217147. [https://www.ncbi.nlm.nih.gov/geo/query/acc.cgi].

Research involving human participants, their data, or biological material

Policy information about studies with human participants or human data. See also policy information about sex, gender (identity/presentation), and sexual orientation and race, ethnicity and racism.

Reporting on sex and gender	NA
Reporting on race, ethnicity, or other socially relevant groupings	NA
Population characteristics	NA
Recruitment	NA
Ethics oversight	NA

Note that full information on the approval of the study protocol must also be provided in the manuscript.

Field-specific reporting

Please select the one below that is the best fit for your research. If you are not sure, read the appropriate sections before making your selection.

☒ Life sciences ☐ Behavioural & social sciences ☐ Ecological, evolutionary & environmental sciences

For a reference copy of the document with all sections, see nature.com/documents/nr-reporting-summary-flat.pdf

Life sciences study design

All studies must disclose on these points even when the disclosure is negative.

Sample size	No statistic tests were used to predetermine samples size. We follow the standards in the field when choosing sample size for in vitro and in vivo experiments ((Xu et al, Nature 612, 564-572, 2022); (Hu et al, Immunity 48, 227-242, 2018); (Ramirez et al, Science Immunology 7, eabj9836, 2022)). For Hi-C, PLAC-seq and RNA-seq experiments, at least 2 biological replicates per group/condition were used to ensure the difference was reproducible. To assess the lymphproferative phenotype of Foxp3 DSM mutant mice, at least 5 mice per group were used.
Data exclusions	No data was excluded from analyses
Replication	Independent expreiment and biological replicate were used to ensure the reproducibility of the results as indicated in the figure legend.
Randomization	Samples or mice were grouped according to treatment or genotypes, and thus not randomized.
Blinding	The histology analyses of tissues was performed blindly by a pathologist. For experiments directly comparing groups of different genotypes (e.g. WT vs DSM) or treatments (e.g. Tcon vs Treg), blinding was not applied because genotypes need to be determined before the experiment.

Reporting for specific materials, systems and methods

We require information from authors about some types of materials, experimental systems and methods used in many studies. Here, indicate whether each material, system or method listed is relevant to your study. If you are not sure if a list item applies to your research, read the appropriate section before selecting a response.

Materials & experimental systems

n/a	Involved in the study
☐	☒ Antibodies
☐	☒ Eukaryotic cell lines
☒	☐ Palaeontology and archaeology
☐	☒ Animals and other organisms
☒	☐ Clinical data
☒	☐ Dual use research of concern
☒	☐ Plants

Methods

n/a	Involved in the study
☐	☒ ChIP-seq
☐	☒ Flow cytometry
☒	☐ MRI-based neuroimaging

Antibodies

Antibodies used

Target protein, Antibody source, Catalog# , Clone , Application dilution
CD45.2-Alexa 700, Biolegend, 109822, 104, Flow (1:400)
CD45RB-APC , eBioscience, 17-0455-81 , C363.16A, Flow (1:400)
TCRb-PE-Cy7 eBioscience 25-5961-82 H57-597 Flow (1:400)
TCRgd-PE , BioLegend, 118108, GL3, Flow (1:400)
CD3-BV650, BioLegend, 100229 , 17A2 , Flow (1:400)
CD4-PerCP-Cy5.5 , TONBO 65-0042-U100, RM4-5, Flow (1:400)
CD8-BV510 , Biolegend, 100752 , 53-6.7, Flow (1:400)
CD25-FITC , TONBO, 35-0251-U100 , PC61.5, Flow (1:400)
CD44-BV650, Biolegend , 103049, IM7 , Flow (1:400)
CD62L-BV605, Biolegend, 104438, MEL-14 , Flow (1:400)
IFNg-APC, eBioscience, 17-7311-82, XMG1.2, Flow (1:400)
IL-5-PE , eBioscience, 12-7052-82, TRFK5, Flow (1:400)
Il-13- eflour660, eBioscience, 50-7133-82, eBio13A Flow (1:400)
IL-17-PE, eBioscience, 12-7177-81 , eBio17B7, Flow (1:400)
human CD2-PE, eBioscience, 12-0021-83, RPA-2.10, Flow (1:200)
NGFR-APC , Biolegend, 345108 , ME20.4, Flow (1:400)
Ghost Viability Dye , TONBO, 13-0865-T100 , Flow (1:1000)
CTCF Millipore 07-729, ChIP (3ug/10million cells)
Foxp3 Rabbit polyclonal antibody generated in house ChIP (5ug/10million cells)

Validation

All the commercial antibodies were validated as stated by the suppliers in their product datasheet, and can be easiliy accessed by using the catalog number and vendor information provided above.
Foxp3 Rabbit polyclonal antibody has been validate in our previous study (Loo et al, Immunity 53, 143-157 (2020))

Eukaryotic cell lines

Policy information about cell lines and Sex and Gender in Research

Cell line source(s)

HEK293T (ATCC #CRL-11268)

Authentication

The identities of HEK293T were frequently checked by their morphological features but has not been authenticated by short tandem repeat (STR) profiling.

Mycoplasma contamination

HEK293T cells were tested negative for mycoplasma contamination

Commonly misidentified lines (See ICLAC register)

No commonly misidentified cell lines were used in this study

Animals and other research organisms

Policy information about studies involving animals; ARRIVE guidelines recommended for reporting animal research, and Sex and Gender in Research

Laboratory animals

All mice used in the present study are in the C57BL/6 genetic background. Rag1–/– mice purchased from the Jackson Laboratory were used for adoptive Treg cell transfer. 6-12 week-old mice were used for the study. Foxp3-Thy1.1 reporter mice, Foxp3-GFP reporter mice, and Foxp3-GFPKO mice were used to isolate T cell populations from the thymus and Tcon and Treg cells from spleen for in-situ Hi-C, PLAC-seq, CUT&RUN, and RNA-seq experiments. Rosa-Cas9/Foxp3Thy1.1 mice were used to isolate Treg cells for CRISPR validation of the effect of Foxp3-binding sites on Helios expression.

Wild animals

The study did not involve wild animals.

Reporting on sex	The study did not consider sex except isolating GFP+Foxp3– Treg- “wannabe” cells from healthy heterozygous female Foxp3GFP-KIKO/WT mice. In the KIKO/WT female mice, about half of the Tregs are WT Tregs, which is sufficient to maintain immune system homeostasis. Therefore, the differences we observed between Treg and Treg wannabes were cell intrinsic, and not due to inflammatory conditions.
Field-collected samples	The study did not involve samples collected from field.
Ethics oversight	Animal experiments were performed under the regulation of the Institutional Animal Care and Use Committee according to the Salk Institute's guidelines

Note that full information on the approval of the study protocol must also be provided in the manuscript.

Plants

Seed stocks	NA
Novel plant genotypes	NA
Authentication	NA

ChIP-seq

Data deposition

☒ Confirm that both raw and final processed data have been deposited in a public database such as GEO.

☐ Confirm that you have deposited or provided access to graph files (e.g. BED files) for the called peaks.

Data access links<br>*May remain private before publication.*	The sequencing data from the RNA-seq, ChIP-seq, CUT&RUN, Hi-C, and PLAC-seq experiments have been deposited in Gene Expression Omnibus (GEO) with the following accession ID: GSE217147. [https://www.ncbi.nlm.nih.gov/geo/query/acc.cgi]. Please use the reviewer token “otkxesceztgvvkz”.
Files in database submission	CD4 T-cell, CTCF ChIP-seq<br>CD4 T-cell, input<br>Treg, CTCF ChIP-seq<br>Treg, input
Genome browser session<br>(e.g. UCSC)	http://genome.ucsc.edu/cgi-bin/hgTracks?db=mm9&lastVirtModeType=default&lastVirtModeExtraState=&virtModeType=default&virtMode=0&nonVirtPosition=&position=chr1%3A69500000%2D69900000&hgsid=1667800510_qFr6E9AvrDTFoncOeLfAH9bGsgAn

Methodology

Replicates	we performed one replicate ChIP experiment
Sequencing depth	ChIP-seq experiments were sequenced to a similar depth, with all ChIP and input samples obtaining between 15-22 million reads.
Antibodies	CTCF antibody was from Millipore, Rabbit anti-CTCF polyclonal antibody. Catalog #07-729
Peak calling parameters	Peak calling was conducted using the default parameters of the “callpeak” function against the mouse genome using macs2 with the matched input sample included as a control.
Data quality	Data quality for ChIP-seq of CTCF was conducted by visual examination of peaks, by analysis for enrichment of the known CTCF motif, and by comparison with co-binding of known CTCF interacting partners (Cohesin).
Software	Software - Reads were aligned using BWA MEM to the mm9 genome. Peaks were called with MACS2.

Flow Cytometry

Plots

Confirm that:

☒ The axis labels state the marker and fluorochrome used (e.g. CD4-FITC).

☒ The axis scales are clearly visible. Include numbers along axes only for bottom left plot of group (a 'group' is an analysis of identical markers).

☒ All plots are contour plots with outliers or pseudocolor plots.

☒ A numerical value for number of cells or percentage (with statistics) is provided.

Methodology

Sample preparation	Single cell suspension was generated from the spleen and thymus. DN, DP, CD8SP, CD4SP, CD25+ Treg precursor, Foxp3lo Treg precursor, thymic Treg cells were isolated by FACS sorting from a thymocyte suspension. Tcon and Treg cells were isolated from the spleen by pre-enrichment with EasySep Mouse CD4+ T cell Isolation Kit (STEMCELL Technologies, Cat# 19852), and FACS sorting.<br><br>To analyze immune cell compositions in Foxp3 DSM mice, a single cell suspension was prepared from the spleen or lymph nodes, treated with red cell lysis buffer, and filtered through 70 µm cell strainer. For transcription factor staining, cells were first stained for surface markers, followed by fixation and permeabilization with reagents from the Foxp3/Transcription Factor Staining Buffer Set (eBioscience, 00-5521-00) and incubated with antibodies according to the manufacturer's protocol. For cytokine analysis, cells were stimulated with phorbol 12-myristate 13-acetate (PMA) (50 ng ml-1; Sigma), ionomycin (500 ng ml-1; Sigma) and GolgiStop (BD) for 5 hours. Cells were incubated with cell surface antibodies on ice for 30 min, and then subjected to intracellular staining using Foxp3/Transcription Factor Staining Buffer Set as described above. Samples were run on a BD FACSAria II Flow Cytometer (Becton Dickinson) and data were analyzed by FlowJo software (Tree Star)
Instrument	BD FACSAria II
Software	FlowJo v10.7.1
Cell population abundance	T cells isolated from the spleen and thymus by FACS sorting were always re-checked for purity (at least 94%) by FACS analysis before downstream experiments.
Gating strategy	Dead cell and doublets cell exclusion criteria were applied to all cell population analyses. For immune cells, all hematopoietic cells were firsted pre-gated as CD45.2+. T cell subsets were then identified using the following markers. DN: CD45+CD4-CD8-; DP:CD45+CD4+CD8+; CD8SP: CD45+CD4-CD8+; CD4SP: CD45+CD4+CD8-CD25- Foxp3-reporter-; CD25+ Treg precursor: CD45.2+CD4+CD8-CD25+Foxp3-reporter-; Foxp3lo Treg precursor: CD45+CD4+CD8-CD25-Foxp3-reporterlow; mature thymic Treg: CD45+CD4+CD8-CD25+Foxp3-reporter+; Splenic Tcon cells: CD45+TCRb+CD4+CD8-Foxp3-reporter- ; splenic Treg cells: CD45+ TCRb+CD4+CD8-Foxp3-reporter (Thy1.1 or GFP)+.

☒ Tick this box to confirm that a figure exemplifying the gating strategy is provided in the Supplementary Information.
